# The effect of analgesics on stimulus evoked pain-like behaviour in animal models for chemotherapy induced peripheral neuropathy- a meta-analysis

**DOI:** 10.1038/s41598-019-54152-8

**Published:** 2019-11-26

**Authors:** Carlijn R. Hooijmans, Derk Draper, Mehmet Ergün, Gert Jan Scheffer

**Affiliations:** 1 Department of Anesthesiology, Pain and Palliative Medicine, Radboud University Medical Center, Nijmegen, The Netherlands; 2 Department for Health Evidence unit SYRCLE, Radboud University Medical Center, Nijmegen, The Netherlands

**Keywords:** Chemotherapy, Experimental models of disease, Translational research

## Abstract

Chemotherapy induced painful peripheral neuropathy (CIPN) is a common dose-limiting side effect of several chemotherapeutic agents. Despite large amounts of human and animal studies, there is no sufficiently effective pharmacological treatment for CIPN. Although reducing pain is often a focus of CIPN treatment, remarkably few analgesics have been tested for this indication in clinical trials. We conducted a systematic review and meta-analyses regarding the effects of analgesics on stimulus evoked pain-like behaviour during CIPN in animal models. This will form a scientific basis for the development of prospective human clinical trials. A comprehensive search identified forty-six studies. Risk of bias (RoB) analyses revealed that the design and conduct of the included experiments were poorly reported, and therefore RoB was unclear in most studies. Meta-analyses showed that administration of analgesics significantly increases pain threshold for mechanical (SMD: 1.68 [1.41; 1.82]) and cold (SMD: 1. 41 [0.99; 1.83]) evoked pain. Subgroup analyses revealed that dexmedetomidine, celecoxib, fentanyl, morphine, oxycodone and tramadol increased the pain threshold for mechanically evoked pain, and lidocaine and morphine for cold evoked pain. Altogether, this meta-analysis shows that there is ground to investigate the use of morphine in clinical trials. Lidocaine, dexmedetomidine, celecoxib, fentanyl, oxycodone and tramadol might be good alternatives, but more animal-based research is necessary.

## Introduction

Chemotherapy induced painful peripheral neuropathy is a common dose-limiting side effect of several chemotherapeutic agents (e.g. taxanes, platinum compounds, vinca alkaloids, epothilones, protease inhibitors and thalidomide). The pathophysiology of chemotherapy induced painful peripheral neuropathy, however, varies depending on which chemotherapeutic agent is being studied^1^.

The prevalence of chemotherapy induced painful peripheral neuropathy appears to be as high as 68% when measured in the first month after chemotherapy^2^.

CIPN often presents itself with impairments in sensory, motor, and sometimes autonomic function. The somatosensory symptoms, often characterised as “neuropathic pain”, affect bilaterally hands and feet (stocking and glove distribution) and can include numbness, tingling sensation, spontaneous burning pain, and hypersensitivity to various stimuli.

Symptoms may occur at any time during the course of chemotherapy or long after the treatment ended. Factors that influence the risk and severity of CIPN include cumulative dose, duration of treatment, combination of multiple neurotoxic chemotherapeutics.

Neuropathic pain, especially in cases where patients develop an acute pain syndrome, lead to dose reduction or early cessation of chemotherapy, thereby potentially impacting patient survival and cancer re-emergence.

Despite the large amount of human and experimental studies so far no sufficiently effective (prophylactic) treatment exists^3–5^. One of the reasons for this could be that many of the agents that have been investigated up until now, are medications that look at preventing and treating CIPN because they have demonstrated efficacy in other common neuropathic pain conditions (such as diabetic neuropathy, and postherpetic neuralgia). This has been done even though CIPN is very different from other neuropathies.

CIPN is often treated with anticonvulsants, antidepressant and opioids^6^. However, a recent systematic review regarding treatment of chemotherapy induced peripheral neuropathy showed only moderate benefit for the antidepressant duloxetine. Other drugs were either not effective (lamotrigine and topical ketamine-amitryptiline) or no conclusions could be drawn due to insufficient level of evidence^7^.

From this systematic review by Hou *et al*. it becomes clear that only very few analgesics have been tested in clinical trials (oxycodone^8^, IV infusion of lidocaine^9^, topical amitryptiline and ketamine^10,11^ of which one is also combined with baclofen^10^. Currently there is only one ongoing randomised clinical trial regarding the effects of lidocaine on CIPN registered at www.clinicaltrials.gov.

Thus, analgesics such as morphine, fentanyl or tramadol, paracetamol, celecoxib, dexmedetomidine have never been tested in CIPN patients, and for analgesics that have been tested in clinical trials there is insufficient evidence to show any efficacy.

Clinicians are reluctant to use opioids in patients suffering from neuropathic pain due to risks relating to tolerance, physical dependence, and unwanted side effects^12^. There are, nevertheless, very few treatment options for chemotherapy induced painful peripheral neuropathy. Further research and evaluation of these drugs seems warranted.

Given the lack of sufficient clinical evidence, a first step in this process should be to rigorously assess all relevant animal evidence concerning the effect of various analgesics on chemotherapy induced painful peripheral neuropathy, before beginning new studies in patients. Such an analysis can subsequently guide further design of clinical trials.

Therefore, in this paper, we have conducted a systematic review regarding the effects of analgesics on behavioural outcomes related to stimulus evoked pain-like behaviour during CIPN in animals to obtain insight in possible promising therapies to be investigated in clinical trials.

Because pain cannot be directly measured in animals, we focussed on behavioural outcomes related to stimulus evoked pain-like behaviour.

## Methods

This systematic review investigated the effects of analgesics on behavioural outcomes related to stimulus evoked pain-like behaviour during CIPN in animals. The review methodology was specified in advance and documented using SYRCLE’s systematic review protocol for animal intervention studies^13^ and put online on the SYRCLE Web site.

### Paper identification and selection

This study used a recently developed database containing all CIPN studies published in PubMed and Embase until 19^th^ of December 2017 (n = 650).

The search strategy is published in Gadgil *et al*.^14^.

Studies were included in this database if they met all of the following criteria: (1) the study was an original full paper which presented unique data; (2) the study was performed in animals *in vivo*; (3) the study examined the effect of chemotherapy (all types); (4) the study reported on the outcome (peripheral) neuropathy (e.g. mechanical allodynia, thermal hyper and hypoalgesia, sensory-motor coordination, electrophysiological measurements and/or histological damage to the peripheral nervous system); (5) the study included an appropriate control group (either an untreated, vehicle treated or placebo treated animal). No language or publication date restrictions were applied.

For this systematic review all papers in this database investigating the effect of analgesics on CIPN were selected. The studies were included if they met the following criteria: (1) a controlled study investigating the effect of analgesics that are used in clinical practice (www.farmacotherapeutischkompas.nl) on CIPN, (2) no combination therapy was used (except for combinations of various analgesic drugs) (3); one of the following outcomes was investigated; mechanical, cold or heat evoked pain like behavior.

Early Review Organising Software (EROS; Institute of Clinical Effectiveness and Health Policy, Buenos Aires, Argentina) was used to randomly allocate the included references of the database to two independent reviewers, who screened it for inclusion based on its title and abstract (DD and CH).

Full-text copies of all publications eligible for inclusion were subsequently assessed by two independent reviewers (CH and DD) and included when they met our pre-specified inclusion criteria. Disagreement was solved by discussion.

### Study characteristics and data extraction

We extracted bibliographical data and study characteristics of the included papers.

The bibliographical details were: author, title, year of publication, journal of publication.

Regarding the actual design of the animal experiment we extracted: species, strain, sex, weight/age, method of induction of CIPN [type of chemotherapy, administration route, dose, frequency, duration of treatment and dosing schedule], administration details of analgesics used (route, dose, frequency, duration of treatment, dosing schedule and timing relative to CIPN induction), type of control, outcome measures (all outcomes related to mechanical, cold and heat evoked pain like behavior).

In each publication, we identified all independent comparisons of all behavioural outcomes related to pain. Subsequently group averages (mean, median or incidence), standard deviation (SD), standard error (SE) or ranges and number of animals per group (n) were reported or could be recalculated.

When data were only presented graphically, they were measured using Universal Desktop Ruler software (http://avpsoft.com/products/udruler/) by two independent reviewers. When outcome measure data were missing, we attempted to contact authors for additional information (a maximum of two emails were sent). When the data could not be obtained, a conservative estimate was used if possible.

### Data -analysis

For investigating the efficacy of analgesics on behavioural outcomes related to stimulus evoked pain-like behaviour during CIPN, Comprehensive Meta-Analysis (CMA version 3) was used.

First, we calculated the effect size (Hedges g) for each individual comparison. When the group size was reported as a range (e.g., 6–9), the lowest number of animals was used in our meta-analysis. When no conservative estimate could be made, the comparison was excluded from the analysis.

When multiple experimental groups were compared to the same control group, the group size of the control group was corrected for the number of comparisons made (n/number of comparisons).

Subsequently we conducted meta-analyses. Despite anticipated heterogeneity, the individual effect sizes were pooled to obtain an overall hedges G and a 95% confidence interval (CI). We used the random effects model^15^, which takes into account the precision of individual studies and the variation between studies and weights each study accordingly.

In case of repeated measures in the intervention group (for example measurements after 30 and 60 min), the largest effect size was selected for each comparison. In case a specific outcome was assessed by different methods (e.g. mechanical allodynia measured with paw pressure and von Frey), only the test with the largest effect size was included in the meta-analysis. In case of wash-out studies (studies in which one group of animals received both the control as different treatments) the number of animals in both the control and experimental group were divided by the number of times a group was used.

^I2^ was used to determine the level of between study heterogeneity.

Subgroups were predefined and registered in a protocol (see Supplemental File 1). Subgroup analyses were planned for species, sex, type of CIPN induction, type of analgesic used and administration.

The results of subgroup analyses were only interpreted when subgroups contained at least data from three independent studies or five comparisons per subgroup.

We expected the variance to be comparable within the subgroups; therefore, we assumed a common among-study variance across subgroups. For subgroup analyses, we adjusted our significance level according to the conservative Bonferroni method to account for multiple analyses (p* number of comparisons). However, differences between subgroups should be interpreted with caution and should only be used for constructing new hypotheses rather than for drawing final conclusions.

In a second analyses, we analysed the effects of analgesics within the first 24 hours after first administration. Outcome data were divided into time periods of one hour (1–60, 61–120 etc) and data were pooled if there was more than one measurement per hour.

To determine the robustness of the analyses, sensitivity analysis was conducted. First the effect of the method used to evaluate the acute effects of analgesics was investigated. Instead of pooling all measurements in 60 minutes the time period was changed to 90 minutes.

Secondly, we assessed the effect of in and excluding the conservatively estimated measurements.

### Risk of bias

We used the SYRCLE Risk of Bias tool^16^ to assess the risk of bias in the included studies.

Two independent reviewers assessed the risk of bias in each included paper (DD and CH).

A ‘yes’ score indicates low risk of bias; a ‘no’ score indicates high risk of bias; and a ‘?’ score indicates unknown risk of bias.

To overcome the problem of judging too many items as “unclear risk of bias” because reporting of experimental details on animals, methods and materials is generally very poor^17,18^ we added two items on reporting: reporting of any measure of randomization, reporting of any measure of blinding. For these two items, a ‘yes’ score indicates ‘reported’, and a ‘no’ score indicates ‘unreported’.

### Publication bias

We used funnel plots and Trim and Fill analysis to search for evidence of publication bias. Because SMDs may cause funnelplot distortion we plotted the SMD against a sample size-based precision estimate (1/√(n))^19^.

## Results

### Study selection

**Figure 1:**
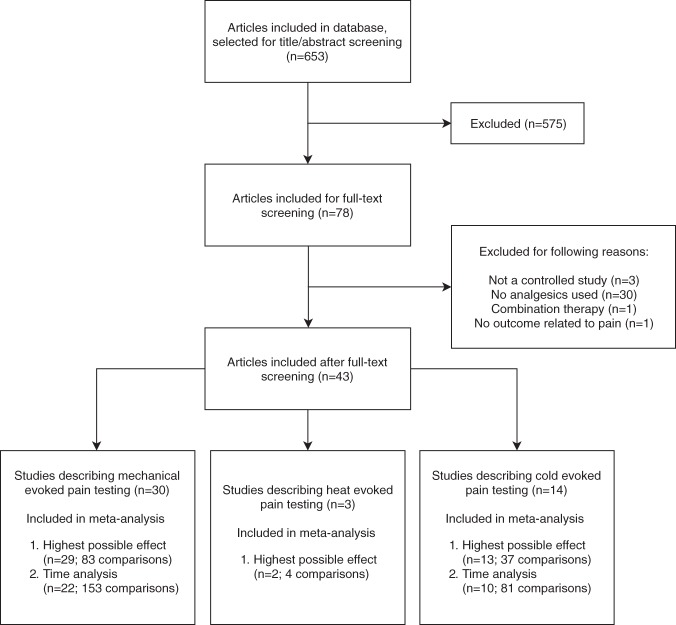
Flow chart of the study selection process.

Figure 1 shows the flow chart of our study selection process. 653 abstracts were screened of which 43^20–62^ were initially included in this review. Out of these 43 studies, 2 were excluded^43,46^ because of unclear description of the number of included comparisons.

In total 124 comparisons coming from 41 independent references, could be included in the systematic review.

### Study characteristics

The characteristics of the included studies are visualised in Table 1.

**Table 1:** Characteristics table.

Reference	Animal Model					Intervention					Outcomes		
ID	Species	Strain	Sex	Chemotherapy	Route	Type	Route	Dose	Frequency	Duration	Mechanical	Cold	Heat
Guindon, 2013_1	Rat	SD	M	Cisplatin	i.p.	Morphine	i.p.	6 mg/kg	Single dose	n.a.	VF	APW	
Deuis, 2014_1	Mice	C57BL/6 J	M	Oxaliplatin	s.c.	Fentanyl	i.p.	0.2 mg/kg	Single dose	n.a.		CP	
Deuis, 2014_2	Mice	C57BL/6 J	M	Cisplatin	s.c.	Fentanyl	i.p.	0.2 mg/kg	Single dose	n.a.	VF		
Park, 2012_1	Rat	SD	M	Vincristine	i.p.	DexmedeTImidine	i.p.	12.5 μg/kg	Single dose	n.a.	VF	APW	
Park, 2012_2	Rat	SD	M	Vincristine	i.p.	DexmedeTImidine	i.p.	25 μg/kg	Single dose	n.a.	VF	APW	
Park, 2012_3	Rat	SD	M	Vincristine	i.p.	DexmedeTImidine	i.p.	50 μg/kg	Single dose	n.a.	VF	APW	
Park, 2012_4	Rat	SD	M	Vincristine	i.p.	DexmedeTImidine	i.p.	100 μg/kg	Single dose	n.a.	VF	APW	
Park, 2010_1	Rat	SD	M	Vincristine	i.p.	Morphine	i.p.	2.5 mg/kg	Single dose	n.a.	VF		
Park, 2010_2	Rat	SD	M	Vincristine	i.p.	Morphine	i.p.	5 mg/kg	Single dose	n.a.	VF		
Park, 2010_3	Rat	SD	M	Vincristine	i.p.	Morphine	i.p.	10 mg/kg	Single dose	n.a.	VF		
Pascual, 2010_1	Rat	Wistar	M	Paclitaxel	i.p.	Morphine	i.p.	1 mg/kg	Single dose	n.a.	VF		RH
Pascual, 2010_2	Rat	Wistar	M	Paclitaxel	i.p.	Morphine	i.p.	2.5 mg/kg	Single dose	n.a.	VF		RH
Pascual, 2010_3	Rat	Wistar	M	Paclitaxel	i.p.	Morphine	i.p.	5 mg/kg	Single dose	n.a.	VF		RH
Pascual, 2010_4	Rat	Wistar	M	Paclitaxel	i.p.	Morphine	i.p.	10 mg/kg	Single dose	n.a.	VF		RH
Pascual, 2010_5	Rat	Wistar	M	Paclitaxel	i.p.	Ketamine	i.p.	12.5 mg/kg	Single dose	n.a.	VF		RH
Pascual, 2010_6	Rat	Wistar	M	Paclitaxel	i.p.	Ketamine	i.p.	25 mg/kg	Single dose	n.a.	VF		RH
Pascual, 2010_7	Rat	Wistar	M	Paclitaxel	i.p.	Ketamine	i.p.	50 mg/kg	Single dose	n.a.	VF		RH
Pascual, 2010_8	Rat	Wistar	M	Paclitaxel	i.p.	Methadone	i.p.	2.5 mg/kg	Single dose	n.a.	VF		RH
Pascual, 2010_9	Rat	Wistar	M	Paclitaxel	i.p.	Methadone	i.p.	5 mg/kg	Single dose	n.a.	VF		RH
YamamoTI, 2015_1	Rat	SD	M	Bortezomib	i.p.	Tramadol	p.o.	1 mg/kg	Single dose	n.a.	VF		
YamamoTI, 2015_2	Rat	SD	M	Bortezomib	i.p.	Tramadol	p.o.	3 mg/kg	Single dose	n.a.	VF		
YamamoTI, 2015_3	Rat	SD	M	Bortezomib	i.p.	Tramadol	p.o.	10 mg/kg	Single dose	n.a.	VF		
YamamoTI, 2015_4	Rat	SD	M	Bortezomib	i.p.	Diclofenac	p.o.	3 mg/kg	Single dose	n.a.	VF		
YamamoTI, 2015_5	Rat	SD	M	Bortezomib	i.p.	Diclofenac	p.o.	10 mg/kg	Single dose	n.a.	VF		
YamamoTI, 2015_6	Rat	SD	M	Bortezomib	i.p.	Diclofenac	p.o.	30 mg/kg	Single dose	n.a.	VF		
Ling, 2007_1	Rat	SD	M	Oxaliplatin	i.v.	Morphine	i.v.	1 mg/kg	Single dose	n.a.		TI	
Ling, 2007_2	Rat	SD	M	Oxaliplatin	i.v.	Morphine	i.v.	2 mg/kg	Single dose	n.a.		TI	
Ling, 2007_3	Rat	SD	M	Oxaliplatin	i.v.	Morphine	i.v.	4 mg/kg	Single dose	n.a.		TI	
Ling, 2007_4	Rat	SD	M	Oxaliplatin	i.v.	Lidocaine	i.v.	1 mg/kg	Single dose	n.a.		TI	
Ling, 2007_5	Rat	SD	M	Oxaliplatin	i.v.	Lidocaine	i.v.	3 mg/kg	Single dose	n.a.		TI	
Ling, 2007_6	Rat	SD	M	Oxaliplatin	i.v.	Lidocaine	i.v.	6 mg/kg	Single dose	n.a.		TI	
Jiang, 2016_1	Mice	C57BL/6 J	M	Oxaliplatin	i.p.	Celecoxib	p.o.	10 mg/kg	Twice daily	13 days	VF	TI	
Jiang, 2016_2	Mice	C57BL/6 J	M	Oxaliplatin	i.p.	Celecoxib	p.o.	30 mg/kg	Twice daily	13 days	VF	TI	
Kim, 2016_1	Mice	C57BL/6	M	Oxaliplatin	i.p.	Morphine	i.p.	0.5 mg/kg	Single dose	n.a.	VF	APW	
Kim, 2016_2	Mice	C57BL/6	M	Oxaliplatin	i.p.	Morphine	i.p.	2 mg/kg	Single dose	n.a.	VF	APW	
Kim, 2016_3	Mice	C57BL/6	M	Oxaliplatin	i.p.	Morphine	i.p.	5 mg/kg	Single dose	n.a.	VF	APW	
Ling, 2008_1	Rat	SD	M	Oxaliplatin	i.p.	Morphine	i.v.	1 mg/kg	Single dose	n.a.		TI	
Ling, 2008_2	Rat	SD	M	Oxaliplatin	i.p.	Morphine	i.v.	2 mg/kg	Single dose	n.a.		TI	
Ling, 2008_3	Rat	SD	M	Oxaliplatin	i.p.	Morphine	i.v.	4 mg/kg	Single dose	n.a.		TI	
Ling, 2008_4	Rat	SD	M	Oxaliplatin	i.p.	Lidocaine	i.v.	1 mg/kg	Single dose	n.a.		TI	
Ling, 2008_5	Rat	SD	M	Oxaliplatin	i.p.	Lidocaine	i.v.	3 mg/kg	Single dose	n.a.		TI	
Ling, 2008_6	Rat	SD	M	Oxaliplatin	i.p.	Lidocaine	i.v.	6 mg/kg	Single dose	n.a.		TI	
Michot, 2014_1	Mice	C57BL/6 J	M	Oxaliplatin	i.p.	Morphine	s.c.	3 mg/kg	Single dose	n.a.	VF		
Michot, 2014_2	Rat	SD	M	Oxaliplatin	i.p.	Morphine	s.c.	3 mg/kg	Single dose	n.a.	VF		
Bujalska, 2008_1	Rat	Wistar	M	Vincristine	i.v.	Celecoxib	s.c.	1 mg/kg	12 day	12 days	PP		
Bujalska, 2008_2	Rat	Wistar	M	Vincristine	i.v.	Indomethacin	s.c.	1 mg/kg	12 day	12 days	PP		
Flatters, 2004_1	Rat	SD	M	Paclitaxel	i.p.	Morphine	i.p	4 mg/kg	Single dose	n.a.	VF		
Flatters, 2004_2	Rat	SD	M	Paclitaxel	i.p.	Morphine	i.p	8 mg/kg	Single dose	n.a.	VF		
ITI, 2012-1	Mice	ddY	M	Paclitaxel	i.p.	ETIdolac	p.o.	10 mg/kg	Daily	14 days	VF		
ITI, 2012-2	Mice	ddY	M	Paclitaxel	i.p.	Indomethacin	p.o.	1 mg/kg	Daily	14 days	VF		
ITI, 2012-3	Mice	ddY	M	Paclitaxel	i.p.	Celecoxib	p.o.	30 mg/kg	Daily	14 days	VF		
ITI, 2012-4	Mice	ddY	M	Paclitaxel	i.p.	Diclofenac	p.o.	3 mg/kg	Daily	14 days	VF		
Zbarcea, 2011_1	Rat	Wistar	M	Paclitaxel	i.p.	Tramadol	p.o.	5 mg/kg	Daily	4 days	VF		
Hidaka, 2009-1	Mice	ddY	M	Paclitaxel	i.p.	loxoprofen	p.o.	1 mg/kg	Daily	5 days	VF		
Balayssac, 2009_1	Rat	SD	M	Cisplatin	i.p.	Morphine		0.5 mg/kg	Single dose	n.a.	PP		
Balayssac, 2009_2	Rat	SD	M	Cisplatin	i.p.	Morphine		2 mg/kg	Single dose	n.a.	PP		
Micheli, 2015_1	Rat	SD	M	Oxaliplatin	i.p.	Morphine	i.t.	0.3 nmol	Single dose	n.a.	PP		
Micheli, 2015_2	Rat	SD	M	Oxaliplatin	i.p.	Morphine	i.t.	3 nmol	Single dose	n.a.	PP		
Micheli, 2015_3	Rat	SD	M	Oxaliplatin	i.p.	Morphine	i.t.	10 nmol	Single dose	n.a.	PP		
Micheli, 2015_4	Rat	SD	M	Paclitaxel	i.p.	Morphine	i.t.	0.3 nmol	Single dose	n.a.	PP		
Micheli, 2015_5	Rat	SD	M	Paclitaxel	i.p.	Morphine	i.t.	3 nmol	Single dose	n.a.	PP		
Micheli, 2015_6	Rat	SD	M	Paclitaxel	i.p.	Morphine	i.t.	10 nmol	Single dose	n.a.	PP		
Bujalska, 2009_1	Rat	Wistar	M	Vincristine	i.v.	Morphine	i.t.	5 mg/kg	Daily	5 days	PP		
Bujalska, 2009_2	Rat	Wistar	M	Vincristine	i.v.	Fentanyl	i.t.	0.0625 mg/kg	Daily	5 days	PP		
Bujalska, 2009_3	Rat	Wistar	M	Vincristine	i.v.	Buprenorphine	i.t.	0.075 mg/kg	Daily	5 days	PP		
Thibault, 2014_1	Rat	SD	M	Vincristine	i.p.	Morphine	i.p.	3.33 mg/kg	Daily	5 days	VF; PT		
Thibault, 2014_2	Rat	SD	M	Vincristine	i.p.	Oxycodone	i.p.	3.33 mg/kg	Daily	5 days	VF; PT		
Kanbara, 2014_1	Rat	SD	M	Oxaliplatin	i.p.	Morphine	i.t.	3 mg/kg	Single dose	n.a.	VF		
Kanbara, 2014_2	Rat	SD	M	Oxaliplatin	i.p.	Fentanyl	i.t.	0.56 mg/kg	Single dose	n.a.	VF		
Kanbara, 2014_3	Rat	SD	M	Oxaliplatin	i.p.	Buprenorphine	i.t.	0.017 mg/kg	Single dose	n.a.	VF		
Park, 2013_1	Mice	C57BL/6	M	Cisplatin	i.p.	Morphine	i.p.	1 mg/kg	Single dose	n.a.	VF		
Park, 2013_2	Mice	C57BL/6	M	Cisplatin	i.p.	Morphine	i.p.	3 mg/kg	Single dose	n.a.	VF		
Park, 2013_3	Mice	C57BL/6	M	Cisplatin	i.p.	Morphine	i.p.	10 mg/kg	Single dose	n.a.	VF		
Park, 2013_4	Mice	C57BL/6	M	Cisplatin	i.p.	KeTIrolac	i.p.	15 mg/kg	Single dose	n.a.	VF		
Zhao, 2014_1	Mice	C57BL/6 J	M	Oxaliplatin	i.p.	Morphine	s.c.	5 mg/kg	Single dose	n.a.		CP	
Zhao, 2014_2	Mice	C57BL/6 J	M	Oxaliplatin	i.p.	Morphine	s.c.	10 mg/kg	Single dose	n.a.		CP	
Zhao, 2014_3	Mice	C57BL/6 J	M	Oxaliplatin	i.p.	Diclofenac	i.p	25 mg/kg	Single dose	n.a.		CP	
Zhao, 2014_4	Mice	C57BL/6 J	M	Oxaliplatin	i.p.	Diclofenac	i.p	50 mg/kg	Single dose	n.a.		CP	
Zhao, 2014_5	Mice	C57BL/6 J	M	Oxaliplatin	i.p.	Tramadol	s.c.	10 mg/kg	Single dose	n.a.		CP	
Zhao, 2014_6	Mice	C57BL/6 J	M	Oxaliplatin	i.p.	Tramadol	s.c.	20 mg/kg	Single dose	n.a.		CP	
Shidahara, 2016_1	Rat	SD	M	Oxaliplatin	i.p.	Tramadol	p.o.	30 mg/kg	Single dose	n.a.		APW	
Kanbara, 2014_1	Rat	SD	M	Oxaliplatin	i.p.	Oxycodone	s.c.	0.1 mg/kg	Single dose	n.a.	VF		
Kanbara, 2014_2	Rat	SD	M	Oxaliplatin	i.p.	Oxycodone	s.c.	0.17 mg/kg	Single dose	n.a.	VF		
Kanbara, 2014_3	Rat	SD	M	Oxaliplatin	i.p.	Oxycodone	s.c.	0.3 mg/kg	Single dose	n.a.	VF		
Kanbara, 2014_4	Rat	SD	M	Oxaliplatin	i.p.	Oxycodone	s.c.	0.56 mg/kg	Single dose	n.a.	VF		
Kanbara, 2014_5	Rat	SD	M	Oxaliplatin	i.p.	Fentanyl	s.c.	0.0056 mg/kg	Single dose	n.a.	VF		
Kanbara, 2014_6	Rat	SD	M	Oxaliplatin	i.p.	Fentanyl	s.c.	0.01 mg/kg	Single dose	n.a.	VF		
Kanbara, 2014_7	Rat	SD	M	Oxaliplatin	i.p.	Fentanyl	s.c.	0.017 mg/kg	Single dose	n.a.	VF		
Kanbara, 2014_8	Rat	SD	M	Oxaliplatin	i.p.	Fentanyl	s.c.	0.056 mg/kg	Single dose	n.a.	VF		
Kanbara, 2014_9	Rat	SD	M	Oxaliplatin	i.p.	Morphine	s.c.	0.3 mg/kg	Single dose	n.a.	VF		
Kanbara, 2014_10	Rat	SD	M	Oxaliplatin	i.p.	Morphine	s.c.	0.56 mg/kg	Single dose	n.a.	VF		
Kanbara, 2014_11	Rat	SD	M	Oxaliplatin	i.p.	Morphine	s.c.	1 mg/kg	Single dose	n.a.	VF		
Kanbara, 2014_12	Rat	SD	M	Oxaliplatin	i.p.	Morphine	s.c.	1.7 mg/kg	Single dose	n.a.	VF		
Kanbara, 2014_13	Rat	SD	M	Oxaliplatin	i.p.	Morphine	s.c.	3 mg/kg	Single dose	n.a.	VF		
Zbarcea, 2011_1	Mice	NMRI	M	Vincristine	i.p.	Tramadol	p.o.	5 mg/kg	Daily	11 days			HP
Nozaki-Taguchi, 2001_1	Rat	SD	M	Vincristine	i.p.	Morphine	i.p.	5 mg/kg	Single dose	n.a.	VF		
Nozaki-Taguchi, 2001_2	Rat	SD	M	Vincristine	i.p.	Lidocaine	i.p.	45 mg/kg	Single dose	n.a.	VF		
Bujalska, 2009_1	Rat	Wistar	M	Vincristine	i.v.	Indomethacin	s.c.	0.1 mg/kg	Daily*	12 days	PP		
Bujalska, 2009_2	Rat	Wistar	M	Vincristine	i.v.	Celecoxib	s.c.	0.1 mg/kg	Daily*	12 days	PP		
Xu, 2011_1	Rat	SD	M	Paclitaxel	i.p.	Morphine	i.p.	5 mg/kg	3 × / weekk	21 days	VF		
Ami, 2012_1	Mice	Crlj:CD1	M	Paclitaxel	i.p.	Morphine	s.c.	0.3 mg/kg	Single dose	n.a.	VF		
Ami, 2012_2	Mice	Crlj:CD1	M	Paclitaxel	i.p.	Morphine	s.c.	1 mg/kg	Single dose	n.a.	VF		
Ami, 2012_3	Mice	Crlj:CD1	M	Paclitaxel	i.p.	Morphine	s.c.	3 mg/kg	Single dose	n.a.	VF		
Ghelardini, 2010_1	Rat	SD	M	Oxaliplatin	i.p.	Tramadol	p.o.	40 mg/kg			PP		
Higuchi, 2015_1	Rat	SD	M	Bortezomib	i.p.	Tramadol	p.o.	10 mg/kg	Single dose	n.a.	VF		
Egashira, 2010_1	Rat	SD	M	Oxaliplatin	i.p.	Lidocaine	i.p.	3 mg/kg	Single dose	n.a.	VF	APW	
Egashira, 2010_2	Rat	SD	M	Oxaliplatin	i.p.	Lidocaine	i.p.	10 mg/kg	Single dose	n.a.	VF	APW	
Egashira, 2010_3	Rat	SD	M	Oxaliplatin	i.p.	Lidocaine	i.p.	30 mg/kg	Single dose	n.a.	VF	APW	
Brusco, 2017_1	Mice	Swiss	M	Paclitaxel	i.p.	Acetaminophen	p.o.	100 mg/kg	Single dose	n.a.	VF		
Brusco, 2017_2	Mice	Swiss	M	Paclitaxel	i.p.	Acetaminophen	p.o.	100 mg/kg	Single dose	n.a.	VF	APW	
Chen, 2016_1	Mice	C57BL/JC	M	Oxaliplatin	i.p.	Celecoxib	p.o.	15 mg/kg	Twice daily	7 days	VF	TI	
Lin, 2017-1	Mice	C57BL/JC	M	Paclitaxel	i.p.	Morphine	i.p.	10 mg/kg	Daily	12 days	VF	APW	
Nie, 2017_1	Rats	SD	M	Vincristine	i.p.	DexmedeTImidine	i.t.	4 μg/kg	Single dose	n.a.	VF		
Nie, 2017_2	Rats	SD	M	Vincristine	i.p.	DexmedeTImidine	i.t.	1.2 μg/kg	Single dose	n.a.	VF		
Nie, 2017_3	Rats	SD	M	Vincristine	i.p.	DexmedeTImidine	i.t.	0.4 μg/kg	Single dose	n.a.	VF		
Parvathy, 2015_1	Mice	BALB/c	M	Paclitaxel	i.p.	Indomethacin	i.p.	10 mg/kg	Single dose	n.a.			HP
Parvathy, 2015_2	Mice	BALB/c	F	Paclitaxel	i.p.	Indomethacin	i.p.	1 mg/kg	Single dose	n.a.			HP
Parvathy, 2015_3	Mice	BALB/c	F	Paclitaxel	i.p.	Indomethacin	i.p.	10 mg/kg	Single dose	n.a.			HP
Salat, 2017_1	Mice	CD-1	M	Oxaliplatin	i.p.	Morphine	s.c.	10 mg/kg	Two doses	n.a.		CP	
Salat, 2017_2	Mice	CD-1	M	Oxaliplatin	i.p.	Cebranopadol	s.c.	10 mg/kg	Two doses	n.a.		CP	
Sanna, 2017_1	Mice	CD-1	M	Oxaliplatin	i.p.	Morphine	i.p.	1 mg/kg	Single dose	n.a.		CP	
Sanna, 2017_2	Mice	CD-1	M	Oxaliplatin	i.p.	Morphine	i.p.	5 mg/kg	Single dose	n.a.		CP	
Han, 2014_1	Rat	SD	M	Cisplatin	i.p.	Morphine	s.c.	0.3, 0.6, 1 mg/kg	Single dose	n.a.	VF		
Han, 2014_2	Rat	SD	M	Cisplatin	i.p.	Mexolicam	i.p.	5, 10, 20 mg/kg	Single dose	n.a.	VF		

Characteristics of all 124 comparisons out of 41 included studies.

SD = Sprague Dawley; M = male; F = female; i.p. = intra peritoneal; i.v. = intra venous; VF = von Frey test; PP = paw pressure test; PT = pinch test; CP = cold plate test; APW = acetone paw withdrawal; TI = tail immersion; RH = radiant heat assay; HP = hot plate test.

All the experiments were conducted in mice (31%) and rats (69%). Only 1 study (containing 3 comparisons) used female animals. Five different chemotherapeutic agents were used for the induction of CIPN: oxaliplatin (44.4%); paclitaxel (24.2%); vincristine (17.7%), cisplatin (8.1%) and bortezomib (5.6%). The dosing schedule and administration route differed considerably between the different animal models (between the different chemotherapies but also within a chemotherapy group).

Eighteen different analgesics were tested (Table 2). Morphine was administered in the majority of comparisons (41.9%). Subsequently, the drugs that were tested most frequently were: lidocaine (8.1%), tramadol (8.1%), fentanyl (6.5%) and dexmedetomidine (5.6%). In the majority of studies, a single dose was administered. The administration route between various analgesics, but also within a group receiving a specific drug varied greatly (Table 2).

**Table 2:** Frequency table of the administered analgesics.

Analgesic	Admin. route	n	%
Acetaminophen	p.o.	2	1.6
Buprenorphine	i.t.	2	1.6
Cebranopadol	s.c.	1	0.8
Celecoxib	p.o.; s.c.	6	4.8
Dexmedetomidine	i.p.; i.t.	7	5.6
Diclofenac	i.p.; p.o.	6	4.8
Etodolac	p.o.	1	0.8
Fentanyl	i.p.; i.t.; s.c.	8	6.5
Indomethacin	i.p.; s.c.; s.c.	6	4.8
Ketamine	i.p.	3	2.4
Ketorolac	i.p.	1	0.8
Lidocaine	i.p. i.v.	10	8.1
loxoprofen	p.o.	1	0.8
Methadone	i.p.	2	1.6
Mexolicam	i.p.	1	0.8
Morphine	i.p.; i.t.; i.v.; s.c.	52	41.9
Oxycodone	i.p.; s.c.	5	4.0
Tramadol	p.o.; s.c.	10	8.1

p.o. = peross/ oral; i.t. = intrathecal; s.c. = subcutaneous; i.p. = intraperitoneal; i.v. = intravenous.

In order to measure stimulus evoked pain-like behaviour, different kind of stimuli (mechanical; heat and cold) were used and analysed. Mechanical stimuli were mainly investigated using the von Frey test and paw pressure test. Only one study conducted the so-called pinch test in which a pincher unit was used to determine the paw withdrawal threshold of the animal.

Pain like behaviour using cold stimuli was mainly investigated using the tail immersion test, cold plate test and acetone test. Heat stimuli were not often used. Three studies used the hot plate test, and one the radiant heat assay.

### Reporting quality and risk of bias

Because reporting of experimental details on animals, methods and materials is generally poor^17^ and as a consequence often many items covered in the risk of bias tool are scored “unclear”, we added two items investigating to the SYRCLE risk of bias tool investigating the quality of reporting; e.g. reporting of any measure of randomization and reporting of any measure of blinding.

**Figure 2:**
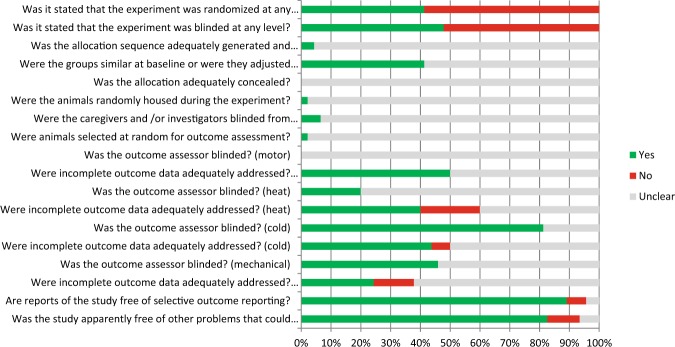
Results of the risk of bias assessment. Results of the risk of bias assessment of the 44 studies included in this systematic review. The first two items assess study quality by scoring reporting, a ‘yes’ score indicating reported, and a ‘no’ score indicating unreported. The other items assessed risk of bias, with ‘yes’ indicating low risk of bias, ‘no’ high risk of bias and ‘?’ unclear risk of bias.

The results of the risk of bias and quality assessment are shown in Fig. 2.

This review clearly revealed that methodological details of animal experiments were often poorly reported. Items related to random housing, random outcome assessment and blinding of caregivers and investigators were rarely described, and therefore scored as an unclear risk of bias. Forty-one percent of the papers included reported measures for randomization of the animals across the experimental groups. However, none of these papers provided information regarding the methods used related to allocation sequence generation.

Forty eight percent of the papers reported measures for blinding. Risk of bias analyses showed that it is the outcome assessor who is predominantly blinded.

Low risk of bias was scored for 89% of the papers for selective outcome reporting. High risk of bias was scored for three papers for the question “Was the study apparently free of other problems that could result in high risk of bias?”. This was due to the fact that one or more of the authors was employed by a manufacturer who investigated medication in a specific study.

## Meta-Analysis

### Stimulus evoked pain-like behaviour

#### Mechanical stimuli

Twentynine studies, containing 83 independent comparisons (938 animals), investigated the effect of administration of analgesics on mechanical evoked pain like behavior in animal models for chemotherapy induced peripheral neuropathy. Overall, administration of analgesics significantly increases the pain threshold (SMD: 1.68 [1.41; 1.82] (n = 83), and thus reduces mechanical evoked pain.

The overall heterogeneity between the studies was moderate (I^2^ = 63%).

Subgroup analyses were only conducted in groups with 3 studies or at least 5 independent comparisons. Consequently, subgroup analyses could only be performed for species, type of chemotherapeutic agent and type of analgesic used.

**Figure 3:**
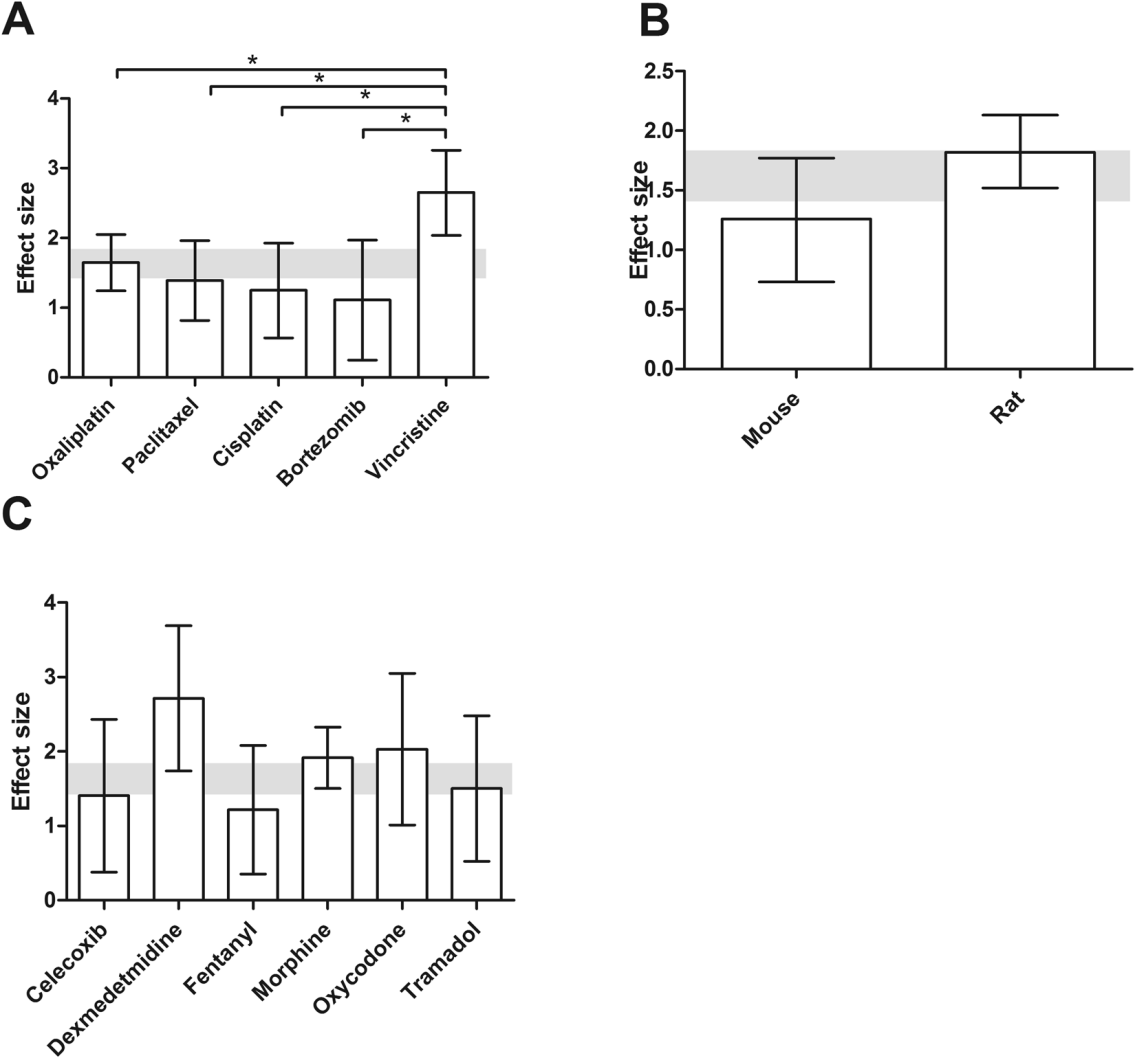
Effect of analgesics on mechanical evoked pain in animal models for chemotherapy induced peripheral neuropathy. (**A**) Type of chemotherapy, (**B**) Animal species, (**C**) Type of analgesic. The grey bars represent the 95% confidence interval of the pooled effect estimate. The columns indicate the effect estimate with the 95% confidence interval of the subgroups. The results from subgroup analyses were only displayed when subgroups contained data of at least 3 studies or 5 independent comparisons.

The reduction of mechanical evoked pain was significantly larger in CIPN models induced with vincristine (SMD2.65 [2.04; 3.26] n = 17)) compared to all other chemotherapeutic agents used (cisplatin (SMD = 1.25 [0.57; 1.93] n = 12) (p = 0.03), bortezomib (SMD = 1.11 [0.25; 1.97] n = 7) (p = 0.04), oxaliplatin (SMD = 1.65 [1.24–2.05] n = 32) (p = 0.05) and paclitaxel (SMD = 1.39 [0.82; 1.96] n = 15) (p = 0.04) (Fig. 3A).

All the other subgroup analyses (species and type of analgesic used) did not show any significant difference between the subgroups (Fig. 3B,C).

Dexmedetomidine (n = 7), celecoxib (n = 5), fentanyl (n = 7), morphine (n = 37), oxycodone (n = 6) and tramadol (n = 6) all increased the pain threshold for mechanical evoked pain (Fig. 3C).

We further investigated the effect of administering analgesics per type of chemotherapy induced CIPN. Five types of chemotherapeutic drugs were used in the included CIPN models (bortezomib (n = 7), paclitaxel (n = 15), oxaliplatin (n = 32), cisplatin (n = 12), vincristine (n = 17).

The effect of morphine was investigated in CIPN models based on cisplatin, oxaliplatin, paclitaxel and vincristine. These models demonstrated that morphine significantly increased the pain threshold (e.g. oxaliplatin (SMD 2.04 [1.40–2.69] n = 14); paclitaxel (SMD 1.92 [1.13–2.71] n = 9), vincristine (SMD 2.27 [1.10–3.44] n = 5) and cisplatin (SMD 1.51 [0.65–2.37] n = 9)). Currently, there are no studies being conducted that investigate the effect of morphine in CIPN models induced with bortezomib.

The effect of fentanyl could only be analyzed in CIPN models induced with oxaliplatin, as the subgroups for the cisplatin and vincristine-based models were too small for meaningful analyses.

Fentanyl (SMD 0.99 [0.02–1.95] n = 5) also significantly increased the pain threshold in CIPN induced with oxaliplatin.

It was only possible to analyze the effect of oxycodon in CIPN models induced with oxaliplatin, as the subgroup for vincristine-based models was too small for meaningful analyses.

Oxycodone (SMD 1.68 [0.63–2.73] n = 5) also significantly increased the pain threshold in CIPN induced with oxaliplatin.

Dexmedetomidine was only tested in vincristine based models and showed significant increases in pain threshold (SMD 2.72 [1.72–3.72] n = 7).

All other drugs tested within a specific CIPN model had subgroups which were too small for further analyses.

#### Time effect

In the second analysis, only studies that measured acute effects (first 24 hours) were included (22 studies containing 153 comparisons). Effect sizes were calculated per hour for the first 4 hours, and thereafter as a large group.

Aadministration of analgesics significantly increased the pain threshold, and therefore reduced mechanical evoked pain, within the first 4 hours after administration (Table 3).

**Table 3:** Acute effects of analgesics on mechanical and cold evoked pain in CIPN models.

	Time	n	ES	LL	UL	p value	I2
Mechanical	1–60 min	70	1.17	0.97	1.37	0.01	65.7
	61–120 min	39	0.75	0.48	1.03	0.000	46.2
	121–180 min	22	0.51	0.13	0.89	0.009	42.1
	181–240 min	15	0.53	0.10	0.97	0.00	0.02
	241 + min	7	0.03	−0.63	0.70	0.92	0.00
Cold	1–60 min	33	0.95	0.74	1.16	0.000	0.00
	61–120 min	23	0.83	0.58	1.07	0.000	26.7
	121–180 min	14	0.59	0.23	0.95	0.001	0.00
	181–240 min	7	0.25	−0.27	0.76	0.35	0.00
	241 + min	4	−0.09	−0.85	0.67	0.82	0.00

N = number of comparisons, ES = effect size/summary effect, LL = lower limit of 95% confidence interval, UL = upper limit of 95% confidence interval, I^2^ = % of between study heterogeneity.

Subgroup effect were only investigated for the subgroups containing at least 3 independent studies or 5 comparisons, and they are listed in Table 4.

**Table 4:** Investigated subgroups in acute effects of analgesics on mechanical and cold evoked pain in CIPN models.

Timing	Subgroup	n
**Mechanical**		
1–60 min	Mice	15
	Rats	55
	Bortezomib	7
	Cisplatin	12
	Oxaliplatin	29
	Paclitaxel	12
	Vincristine	10
	Fentanyl	6
	Morphine	36
	Oxycodone	6
	Tramadol	5
61–120 min	Mice	7
	rats	32
	Bortezomib	7
	Cisplatin	11
	Oxaliplatin	11
	Vincristine	10
	Morphine	21
121–180 min	Mice	7
	Rats	15
	Cisplatin	11
	Oxaliplatin	5
	Vincristine	5
	Morphine	15
181–240 min	Mice	7
	Rats	8
	Cisplatin	6
	Morphine	6
**Cold**		
Timing	Subgroup	n
1–60 min	Mouse	12
	Rat	21
	Oxaliplatin	28
	Lidocaine	9
	Morphine	14
61–120 min	Rat	20
	Oxaliplatin	18
	Lidocaine	9
	Morphine	10
121–180 min	Rat	11
	Oxaliplatin	9
	Lidocaine	6
181–240 min	Rat	7

Number of comparisons (n) per subgroup in the acute effect analysis. Only the subgroups containing at least 5 comparisons are listed here.

Morphine increased the pain threshold in the first (SMD 1.36 [1.06–1.65] n = 36), second (SMD 1.00 [0.66–1.35] n = 21) and third hour (SMD 0.59 [0.15–1.02] n = 15).

### Cold stimuli

Thirteen studies, containing 37 independent comparisons, investigated the effect of administering analgesics on cold evoked pain like behavior in animal models for chemotherapy induced peripheral neuropathy.

Overall, administration of analgesics significantly increased the pain threshold (SMD: 1. 41 [0.99; 1.83] n = 37), and thus reduced mechanical evoked pain.

The overall heterogeneity between the studies was moderate to high (I^2^ = 69%).

**Figure 4:**
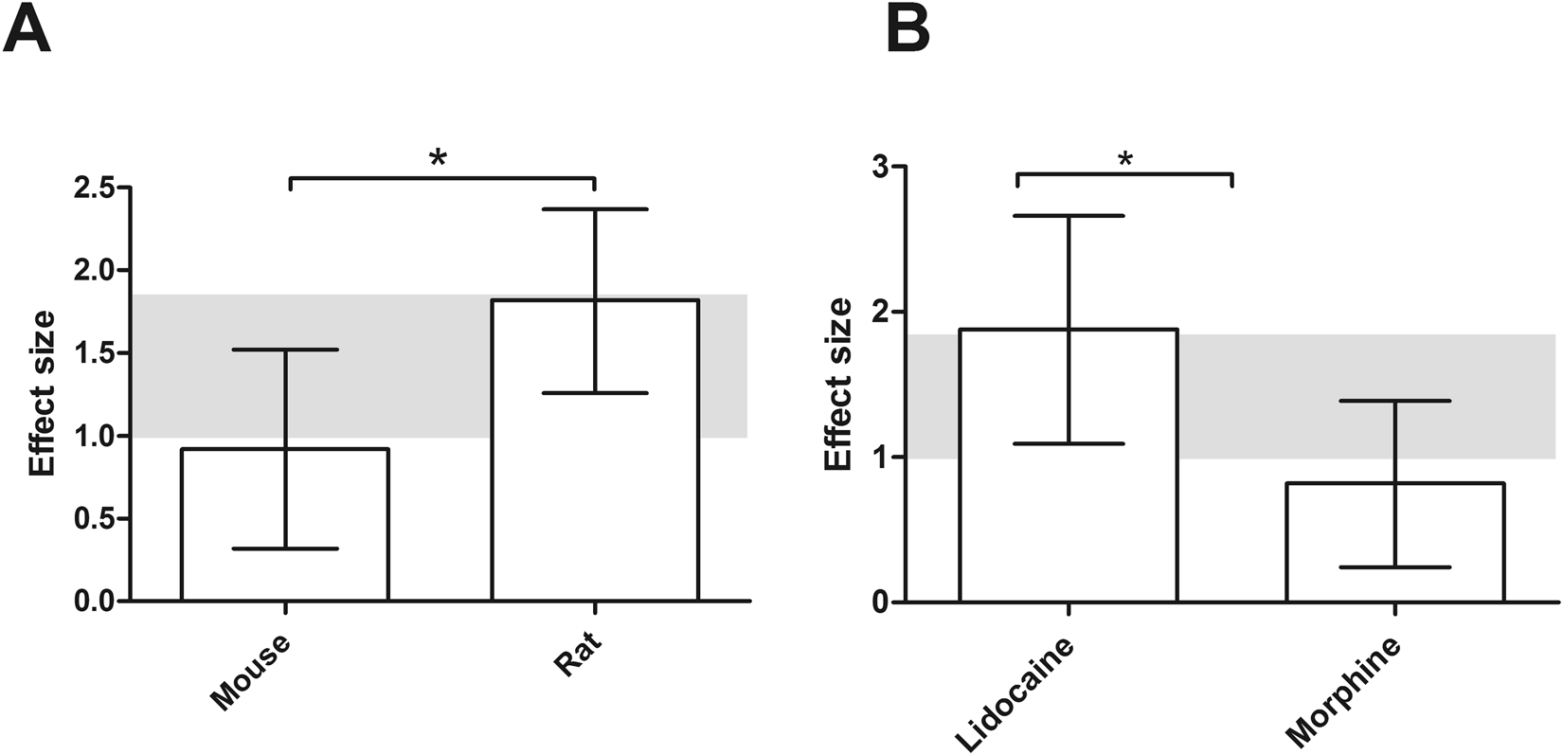
The effect of administration of analgesics on cold evoked pain like behavior in animal models for chemotherapy induced peripheral neuropathy. (**A**) Animal species, (**B**) Type of analgesic. The grey bars represent the 95% confidence interval of the pooled effect estimate. The columns indicate the effect estimate with the 95% confidence interval of the subgroup. The results from subgroup analyses were only displayed when subgroups contained data of at least 3 studies or 5 independent comparisons.

Subgroup analyses revealed that the effect of analgesics was larger (p = 0.04) in rats (SMD = 1.82 [1.26; 2.37] n = 21) than in mice (SMD = 0.92 [0.32; 1.52] n = 16) (Fig. 4A).

Interestingly, the effect of lidocaine (SMD = 1.88 [1.09; 2.66] n = 9) was found to be significantly greater compared to morphine (SMD 0.82 [0.24; 1.39] (p = 0.04) (Fig. 4B).

Subgroup analyses for type of chemotherapy revealed that oxaliplatin was used in the majority of studies (n = 31 out of n = 37). Consequently, we could only investigate the effect of administering analgesics in oxaliplatin induced CIPN. Within the oxaliplatin subgroup six types of analgesics were tested. Only the morphine and lidocaine subgroups were large enough for further analyses.

Lidocaine and morphine significantly increased the pain threshold for cold evoked pain. The effect of lidocaine (SMD 1.84 [1.17–2.52] n = 9) was greater than morphine (SMD 0.96 [0.44–1.47] n = 13) administration (p = 0.05).

#### Time effect

Ten studies containing 81 comparisons were used to study the acute effects of analgesics.

Administering analgesics significantly increases the pain threshold, and therefore reduces cold evoked pain, within the first 3 hours after administration (Table 3).

Subgroup effects were only investigated in the subgroups containing at least 3 independent studies or 5 comparisons. They are listed in Table 4.

Morphine (SMD = 0.87 [0.56; 1.18] n = 14) and lidocaine (SMD = 0.88 [0.49; 1.27] n = 9) both increased pain threshold.

During the second hour after administration, lidocaine (SMD = 1.12 [0.76; 1.49] n = 9) significantly increased the pain threshold (p = 0.04) compared to morphine (SMD = 0.58 [0.24; 0.91] n = 10). All of the analgesic subgroups were too small to conduct meaningful analyses.

It was not possible to conduct a subgroup analyses based on the type of chemotherapeutic agent because there were only enough comparisons for one agent (oxaliplatin).

### Heat stimuli

Only two studies, containing 4 independent comparisons investigated the effect of administering analgesics on heat evoked pain like behavior in animal models for chemotherapy induced peripheral neuropathy.

No effects of analgesics were observed in the heat stimulated pain threshold experiments (SMD = 0.26 [−1.51; 1.03] n = 4).

The overall heterogeneity between the studies was moderate to high (I^2^ = 81%).

Subgroup analyses and analyses of the acute effects of analgesics could not be conducted due to the low number of studies.

### Sensitivity analysis

To assess the robustness of our findings and to further explain the observed study heterogeneity, we performed a sensitivity analysis for some of the decisions we made in this review.

Instead of pooling all measurements within 60 minutes, the time period was changed to 90 minutes. In addition, we looked to see if including the moment with the greatest effect compared to pooling all time points within an hour would significantly alter our conclusions. Our results appeared to be robust. Excluding the conservatively extracted measurements in the first analysis did not change the conclusions either.

### Publication bias

Inspection of the funnel plots for mechanical evoked pain like behavior and cold evoked pain like behavior did not show asymmetry (Supplemental Files 1 and 2). Duval and Tweedie’s Trim and Fill analysis confirmed this and showed no extra data points. This indicates that there was no significant overestimation of the observed effects due to publication bias.

## Discussion

In this paper we conducted a systematic review regarding the effects of analgesics on behavioural outcomes related to stimulus evoked pain-like behaviour (mechanical, cold and heat) during CIPN to obtain insight in possible promising therapies to be investigated in clinical trials.

We showed that administration of analgesics significantly increases the pain threshold for mechanical and cold evoked pain. This relationship was not observed in heat evoked pain.

The effect of analgesics was largest within the first hour after administration, but also still effective within 3–4 hours after administration. The effect of various types of analgesic drugs on mechanical evoked pain was investigated with dexmedetomidine, celecoxib, fentanyl, morphine, oxycodone and tramadol. Each analgesic increased the pain threshold significantly, and we didn’t observe any statistical difference between the various drugs. It was only possible to conduct subgroup analysis using Lidocaine and Morphine for cold evoked pain. Administration of both lidocaine and morphine significantly increased the pain threshold, but the effect of Lidocaine was larger than Morphine. Unfortunately, the number of studies for other types of analgesics was too restricted to conduct a meaningful analysis, and therefore no conclusion about effectivity of those drugs could be drawn.

Based on the current available evidence, morphine seems to be the most effective analgesic treatment as it increases pain threshold for both mechanical and cold evoked pain. Lidocaine, dexmedetomidine, celecoxib, fentanyl, oxycodone and tramadol might be good alternatives as well, as they appeared effective in increasing the pain threshold for either mechanical or cold evoked pain.

Considering that morphine seems to be the most effective analgesic treatment and that there is a large base of evidence (morphine has been investigated in 37 independent experiments/comparisons), suggesting that there is ground to investigate the use of morphine in clinical trials.

Lidocaine, dexmedetomidine, celecoxib, fentanyl, oxycodone and tramadol seem promising as well, however, the evidence base is much smaller and currently there is only evidence that those drugs are effective in increasing the pain threshold for either mechanical or cold evoked pain. It is preferable to conduct more animal-based research with those drugs before taking the step to clinical trials.

One of the benefits of the using opioid analgesics (such as morphine, fentanyl, oxycodone and tramadol) in first-line therapy is the immediate onset of relief offered while titrating to therapeutic dose of TCAs, SNRIs and anticonvulsants^63,64^.

However, although opioids seem effective in treating CIPN there are also some serious concerns regarding long term use of opioids such as morphine. A recent publication from the British Pain Society for example states that ‘patients must be aware of uncertainty regarding the long-term effects of opioids, particularly in relation to endocrine and immune function’^65^.

It is suggested that long term opioid therapy increases the risk of developing tolerance and opioid-induced hyperalgesia^66^. Buprenorphine, a morphine like drug, appears to have a much lower risk for developing addiction and hyperalgesia and does not seem to have an impact on immune function.

More preclinical research into the long-term effects of for example morphine is needed, and into alternative morphine like drugs like buprenorphine (so far we could only identify one study^24^, and this study showed a non-significant decrease in mechanical evoked pain).

The finding that oxycodone might be effective is in line with the only published clinical trial regarding this drug. This study showed that pain intensity significantly decreased on day 14^8^. The confidence in the evidence was, however, rated low^7^, and the duration of the study was very short.

Also, there is some clinical evidence available suggesting efficacy of lidocaine in CIPN. A prospective study by van den Heuvel *et al*. showed that pain scores significantly improved after IV lidocaine infusion^9^. However, the level of evidence scored very low, partly because the study was very small and not controlled.

As before mentioned, the observed effects of analgesics were identified for mechanical evoked and cold evoked pain. Surprisingly, we did not find an effect on the pain threshold for heat evoked pain when analgesics were administered. We hypothesize that this is simply the consequence of very limited data. We could only identify three studies that investigated this outcome of which two studies with in total four comparisons could be included in meta-analysis.

Further, the reduction of mechanical evoked pain was significantly larger in CIPN models induced with vincristine compared to cisplatin, bortezomib, oxaliplatin and paclitaxel. Although analgesics reduce mechanical evoked pain very well, we think however, that this finding is not that important as we know from previous research^14^ that often administration routes for vincristine are used that are not used or even contra-indicated in humans. In this case 12 out of 17 studies administered vincristine i.p. (an administration route not used in humans). By using administration routes that poorly match the clinical situation, construct validity can be threatened.

Scientists should use effective and robust animal models that mimic the clinical situation as much as possible. For the CIPN field we recently published a comparison of all available CIPN models which may help scientists selecting suitable CIPN models for their research^14^.

By using effective and robust animal models that mimic the clinical situation as much as possible, the translational value of preclinical study results with respect to the potential of identifying promising treatments for CIPN in the future, will improve.

Currently we do not know much regarding the mechanism of action in the promising analgesics identified in this review. The mechanism of action of the various analgesics may vary per chemotherapeutic agent used, as the pathogenesis and pathophysiological effects of specific chemotherapeutic agents seems to vary per chemotherapeutic agent (disrupted microtubule-mediated axonal transport, axonal degeneration, direct damage to the dorsal root ganglion, and mitochondrial dysfunction have all been shown in previous studies^1^). An extensive literature review on possible mechanism underlying the promising drug candidates is recommended.

### Limitations

This review has some important limitations. Firstly, we summarize and compare animal models for CIPN based on outcome measures related to evoked allodynia/ hyperalgesia and neurophysiological alterations in nerve function (electrophysiological measurements and/or histological damage to the peripheral nervous system), whereas many patients also report other symptoms such as numbness, tingling and ongoing pain.

Besides the fact that none of the included studies investigated the effect of analgesics in CIPN models on spontaneous pain behaviour (such as the grimace scale, burrowing, weights bearing) nor the neurophysiological alterations in nerve function, it would theoretically also be better to use animal models that replicate all symptoms observed in humans. However, this remains very challenging. Symptoms like numbness, tingling and ongoing pain rely on verbal reporting from the patient, often occur spontaneously, and therefore are very difficult to replicate in animal models. Fortunately, investigation into novel measures of ongoing pain in rodents is emerging, but for now, developing animal models of CIPN which replicate all the symptoms that patients report remains very challenging, and we therefore decided to focus in this review on evoked allodynia/ hyperalgesia and neurophysiological alterations in nerve function.

Secondly, all but one of the studies that were included in this review used male animals. Females are greatly underrepresented. Although this is far from unique across research areas, this is problematic and reduces the construct validity and external validity.

Within the field of neuropathic pain this problem is even more disturbing, as recent research showed sex-specific mechanisms for the development of neuropathic pain^67^ and it would therefore not be surprising if pain-relieving drugs that work for one sex might fail in the other half of the population.

Thirdly, the scope of the current review was to investigate whether or not treatment with analgesics may be a promising treatment strategy for chemotherapy induced peripheral neuropathy, and whether or not analgesic treatment causes immediate pain reducing effects (timing analysis; the effect of analgesics in the first 24 h after the first treatment). The effects of repeated dosing and long-term treatment were beyond the scope of this review but need to be further investigated in future research.

Fourthly, another important issue regarding clinical relevance of the included models is that the majority of animal models was cancer free, whereas in the clinical situation most CIPN patient have or experienced previously cancer which may confound the results related to the used animal models.

Fifthly, our risk of bias analysis revealed that essential details regarding the design and conduct of the included experiments are poorly reported. As a consequence, the risk of bias could not be estimated in the majority of studies. Although this is no exception in this field, it is worrying as lack of reporting important methodological details will to some extent indicate neglected use of these methods to reduce bias causing skewed results^68^ and this may seriously hampers drawing reliable conclusions from the included animal studies.

Sixthly, analyses of the between study heterogeneity levels revealed moderate to severe levels of heterogeneity. Heterogeneity in animal research can be expected, as a result from the often-exploratory approach. In other words, part of the heterogeneity is intentionally induced^69^.

To account for anticipated heterogeneity, we used a random effects model, conducted sensitivity analyses and explored the suggested causes for between study heterogeneity by means of subgroup analyses. Exploring this heterogeneity is one of the added values of meta-analyses of animal studies and might help to inform the design of future animal studies and subsequent clinical trials. Some of our most important findings in this paper, e.g. the efficacy of various types of analgesics in increasing pain threshold, are a consequence of the information we obtained from exploring the sources of heterogeneity.

### Conclusions

This review shows that there is ground to investigate the use of morphine in clinical trials.

Lidocaine, dexmedetomidine, celecoxib, fentanyl, oxycodone and tramadol seem promising as well, but more animal-based research into those drugs is preferred before taking the step to clinical trials. Future animal research should use effective and robust animal models that mimic the clinical situation as much as possible, study both sexes, and focus on the efficacy of lidocaine, dexmedetomidine, celecoxib, fentanyl, oxycodone and tramadol. We further emphasize that there is an urgent need for improving the reporting and methodological quality of the conducted future animal experiments.

## Supplementary information

Supplementary information 

## Data Availability

The data that support the findings of this study available from the corresponding author upon reasonable request.

## References

[1] Addington James, Freimer Miriam (2016). Chemotherapy-induced peripheral neuropathy: an update on the current understanding. F1000Research.

[2] Seretny M (2014). Incidence, prevalence, and predictors of chemotherapy-induced peripheral neuropathy: A systematic review and meta-analysis. Pain.

[3] Hershman DL (2014). Prevention and management of chemotherapy-induced peripheral neuropathy in survivors of adult cancers: American Society of Clinical Oncology clinical practice guideline. Journal of clinical oncology: official journal of the American Society of Clinical Oncology.

[4] Majithia N (2016). National Cancer Institute-supported chemotherapy-induced peripheral neuropathy trials: outcomes and lessons. Supportive care in cancer: official journal of the Multinational Association of Supportive Care in Cancer.

[5] Albers, J., Chaudhry, V., Cavaletti, G. & Donehower, R. Interventions for preventing neuropathy caused by cisplatin and related compounds. *The Cochrane database of systematic reviews*, CD005228, 10.1002/14651858.CD005228.pub2 (2007). 10.1002/14651858.CD005228.pub2 17253547

[6] Kim JH, Dougherty PM, Abdi S (2015). Basic science and clinical management of painful and non-painful chemotherapy-related neuropathy. Gynecol Oncol.

[7] Hou S, Huh B, Kim HK, Kim KH, Abdi S (2018). Treatment of Chemotherapy-Induced Peripheral Neuropathy: Systematic Review and Recommendations. Pain Physician.

[8] Cartoni C (2012). Controlled-release oxycodone for the treatment of bortezomib-induced neuropathic pain in patients with multiple myeloma. Support Care Cancer.

[9] van den Heuvel SAS (2017). Intravenous Lidocaine: Old-School Drug, New Purpose-Reduction of Intractable Pain in Patients with Chemotherapy Induced Peripheral Neuropathy. Pain Res Manag.

[10] Barton DL (2011). A double-blind, placebo-controlled trial of a topical treatment for chemotherapy-induced peripheral neuropathy: NCCTG trial N06CA. Support Care Cancer.

[11] Gewandter JS (2014). A phase III randomized, placebo-controlled study of topical amitriptyline and ketamine for chemotherapy-induced peripheral neuropathy (CIPN): a University of Rochester CCOP study of 462 cancer survivors. Support Care Cancer.

[12] Kaley TJ, Deangelis LM (2009). Therapy of chemotherapy-induced peripheral neuropathy. Br J Haematol.

[13] de Vries RBM (2015). A protocol format for the preparation, registration and publication of systematic reviews of animal intervention studies. Evidence-based Preclinical Medicine.

[14] Hooijmans, C. R., Draper, D., Ergun, M. & Scheffer, G. J. A systematic summary and comparison of animal models for chemotherapy induced (peripheral) neuropathy (CIPN). *PLoS One* (2019). 10.1371/journal.pone.0221787 PMC6713358 31461480

[15] DerSimonian, R. & Laird, N. Meta-analysis in clinical trials. *Control Clin Trials* **7**, 177–188, doi:0197-2456(86)90046-2 [pii] (1986). 10.1016/0197-2456(86)90046-2 3802833

[16] Hooijmans CR (2014). SYRCLE’s risk of bias tool for animal studies. BMC medical research methodology.

[17] Kilkenny C (2009). Survey of the quality of experimental design, statistical analysis and reporting of research using animals. PLoS One.

[18] Avey MT (2016). The Devil Is in the Details: Incomplete Reporting in Preclinical Animal Research. PLoS One.

[19] Zwetsloot, P. P. *et al*. Standardized mean differences cause funnel plot distortion in publication bias assessments. *Elife* **6**, 10.7554/eLife.24260 (2017). 10.7554/eLife.24260 PMC5621838 28884685

[20] Ami N, Okamoto K, Oshima H (2012). Analgesic effect of magnetic stimulation on paclitaxel-induced peripheral neuropathic pain in mice. Brain research.

[21] Balayssac D (2009). Increase in morphine antinociceptive activity by a P-glycoprotein inhibitor in cisplatin-induced neuropathy. Neuroscience letters.

[22] Brusco I (2017). alpha-Spinasterol: a COX inhibitor and a transient receptor potential vanilloid 1 antagonist presents an antinociceptive effect in clinically relevant models of pain in mice. British journal of pharmacology.

[23] Bujalska M, Gumulka SW (2008). Effect of cyclooxygenase and nitric oxide synthase inhibitors on vincristine induced hyperalgesia in rats. Pharmacological reports: PR.

[24] Bujalska M, Makulska-Nowak H (2009). Bradykinin receptor antagonists and cyclooxygenase inhibitors in vincristine- and streptozotocin-induced hyperalgesia. Pharmacological reports: PR.

[25] Bujalska M, Makulska-Nowak H, Gumulka SW (2009). Magnesium ions and opioid agonists in vincristine-induced neuropathy. Pharmacological reports: PR.

[26] Chen H (2016). Celecoxib alleviates oxaliplatin-induced hyperalgesia through inhibition of spinal ERK1/2 signaling. Journal of toxicologic pathology.

[27] Deuis JR (2014). Analgesic effects of clinically used compounds in novel mouse models of polyneuropathy induced by oxaliplatin and cisplatin. Neuro-oncology.

[28] Egashira N (2010). Mexiletine reverses oxaliplatin-induced neuropathic pain in rats. Journal of pharmacological sciences.

[29] Flatters SJ, Bennett GJ (2004). Ethosuximide reverses paclitaxel- and vincristine-induced painful peripheral neuropathy. Pain.

[30] Ghelardini C (2010). Effects of a new potent analog of tocainide on hNav1.7 sodium channels and *in vivo* neuropathic pain models. Neuroscience.

[31] Guindon J, Lai Y, Takacs SM, Bradshaw HB, Hohmann AG (2013). Alterations in endocannabinoid tone following chemotherapy-induced peripheral neuropathy: effects of endocannabinoid deactivation inhibitors targeting fatty-acid amide hydrolase and monoacylglycerol lipase in comparison to reference analgesics following cisplatin treatment. Pharmacological research.

[32] Han FY, Wyse BD, Smith MT (2014). Optimization and pharmacological characterization of a refined cisplatin-induced rat model of peripheral neuropathic pain. Behavioural pharmacology.

[33] Hidaka T (2009). Herbal medicine Shakuyaku-kanzo-to reduces paclitaxel-induced painful peripheral neuropathy in mice. European journal of pain (London, England).

[34] Higuchi H, Yamamoto S, Ushio S, Kawashiri T, Egashira N (2015). Goshajinkigan reduces bortezomib-induced mechanical allodynia in rats: Possible involvement of kappa opioid receptor. Journal of pharmacological sciences.

[35] Ito S (2012). Etodolac, a cyclooxygenase-2 inhibitor, attenuates paclitaxel-induced peripheral neuropathy in a mouse model of mechanical allodynia. The Journal of pharmacology and experimental therapeutics.

[36] Jiang SP (2016). Celecoxib reverts oxaliplatin-induced neuropathic pain through inhibiting PI3K/Akt2 pathway in the mouse dorsal root ganglion. Experimental neurology.

[37] Kanbara T (2014). Morphine and oxycodone, but not fentanyl, exhibit antinociceptive effects mediated by G-protein inwardly rectifying potassium (GIRK) channels in an oxaliplatin-induced neuropathy rat model. Neuroscience letters.

[38] Kanbara T (2014). The contribution of Gi/o protein to opioid antinociception in an oxaliplatin-induced neuropathy rat model. Journal of pharmacological sciences.

[39] Kim W (2016). Combined Effects of Bee Venom Acupuncture and Morphine on Oxaliplatin-Induced Neuropathic Pain in Mice. Toxins.

[40] Lin X, Dhopeshwarkar AS, Huibregtse M, Mackie K, Hohmann AG (2018). Slowly Signaling G Protein-Biased CB2 Cannabinoid Receptor Agonist LY2828360 Suppresses Neuropathic Pain with Sustained Efficacy and Attenuates Morphine Tolerance and Dependence. Molecular pharmacology.

[41] Ling B, Authier N, Balayssac D, Eschalier A, Coudore F (2007). Behavioral and pharmacological description of oxaliplatin-induced painful neuropathy in rat. Pain.

[42] Ling B, Coudore F, Decalonne L, Eschalier A, Authier N (2008). Comparative antiallodynic activity of morphine, pregabalin and lidocaine in a rat model of neuropathic pain produced by one oxaliplatin injection. Neuropharmacology.

[43] Lynch JJ, Wade CL, Zhong CM, Mikusa JP, Honore P (2004). Attenuation of mechanical allodynia by clinically utilized drugs in a rat chemotherapy-induced neuropathic pain model. Pain.

[44] Micheli L (2015). Intrathecal administration of nociceptin/orphanin FQ receptor agonists in rats: A strategy to relieve chemotherapy-induced neuropathic hypersensitivity. European journal of pharmacology.

[45] Michot B, Kayser V, Bastian G, Bourgoin S, Hamon M (2014). Differential pharmacological alleviation of oxaliplatin-induced hyperalgesia/allodynia at cephalic versus extra-cephalic level in rodents. Neuropharmacology.

[46] Mori T (2014). Establishment of opioid-induced rewarding effects under oxaliplatin- and Paclitaxel-induced neuropathy in rats. Journal of pharmacological sciences.

[47] Nie B (2017). Synergistic Interaction Between Dexmedetomidine and Ulinastatin Against Vincristine-Induced Neuropathic Pain in Rats. The journal of pain: official journal of the American Pain Society.

[48] Nozaki-Taguchi N, Chaplan SR, Higuera ES, Ajakwe RC, Yaksh TL (2001). Vincristine-induced allodynia in the rat. Pain.

[49] Park BY, Park SH, Kim WM, Yoon MH, Lee HG (2010). Antinociceptive Effect of Memantine and Morphine on Vincristine-induced Peripheral Neuropathy in Rats. The Korean journal of pain.

[50] Park HJ (2012). Analgesic effects of dexmedetomidine in vincristine-evoked painful neuropathic rats. Journal of Korean medical science.

[51] Park HJ (2013). Persistent hyperalgesia in the cisplatin-treated mouse as defined by threshold measures, the conditioned place preference paradigm, and changes in dorsal root ganglia activated transcription factor 3: the effects of gabapentin, ketorolac, and etanercept. Anesthesia and analgesia.

[52] Parvathy SS, Masocha W (2015). Coadministration of indomethacin and minocycline attenuates established paclitaxel-induced neuropathic thermal hyperalgesia: Involvement of cannabinoid CB1 receptors. Scientific reports.

[53] Pascual D, Goicoechea C, Burgos E, Martin MI (2010). Antinociceptive effect of three common analgesic drugs on peripheral neuropathy induced by paclitaxel in rats. Pharmacology, biochemistry, and behavior.

[54] Salat K, Furgala A, Salat R (2018). Evaluation of cebranopadol, a dually acting nociceptin/orphanin FQ and opioid receptor agonist in mouse models of acute, tonic, and chemotherapy-induced neuropathic pain. Inflammopharmacology.

[55] Sanna MD, Ghelardini C, Galeotti NSt (2017). John’s Wort Potentiates anti-Nociceptive Effects of Morphine in Mice Models of Neuropathic Pain. Pain medicine (Malden, Mass.).

[56] Shidahara Y (2016). Pharmacological comparison of a nonhuman primate and a rat model of oxaliplatin-induced neuropathic cold hypersensitivity. Pharmacology research & perspectives.

[57] Thibault K (2014). Molecular mechanisms underlying the enhanced analgesic effect of oxycodone compared to morphine in chemotherapy-induced neuropathic pain. PloS one.

[58] Xu F (2011). Antinociceptive efficacy of verticinone in murine models of inflammatory pain and paclitaxel induced neuropathic pain. Biological & pharmaceutical bulletin.

[59] Yamamoto S (2015). Behavioral and pharmacological characteristics of bortezomib-induced peripheral neuropathy in rats. Journal of pharmacological sciences.

[60] Zbarcea, C., Negreș, S. & Chirita, C. *Gabapentin, alone and associated with tramadol reduces peripheral paclitaxel-induced neuropathy in rats*. Vol. 59 (2011).

[61] Zbarcea, C., Negreș, S., Nicoleta Cristea, A. & Chirita, C. *The effect of dextromethorphan, gabapentin, amitriptyline and tramadol on a mouse model of vincristine - induced peripheral neuropathy*. Vol. 59 (2011).

[62] Zhao M (2014). Pharmacological characterization of standard analgesics on oxaliplatin-induced acute cold hypersensitivity in mice. Journal of pharmacological sciences.

[63] Dworkin RH (2010). Recommendations for the Pharmacological Management of Neuropathic Pain: An Overview and Literature Update. Mayo Clin Proc.

[64] Dworkin RH (2007). Pharmacologic management of neuropathic pain: Evidence-based recommendations. Pain.

[65] Opioids for persistent pain: summary of guidance on good practice from the British Pain Society. *Br J Pain* **6**, 9–10, 10.1177/2049463712436536 (2012). 10.1177/2049463712436536 PMC4590092 26516460

[66] Angst MS, Clark JD (2006). Opioid-induced hyperalgesia - A qualitative systematic review. Anesthesiology.

[67] North RY (2019). Electrophysiological and transcriptomic correlates of neuropathic pain in human dorsal root ganglion neurons. Brain.

[68] Hirst JA (2014). The need for randomization in animal trials: an overview of systematic reviews. PLoS One.

[69] Hooijmans CR (2018). Facilitating healthcare decisions by assessing the certainty in the evidence from preclinical animal studies. PLoS One.

