# Supplementary material for: The effect of analgesics on stimulus evoked pain-like behaviour in animal models for chemotherapy induced peripheral neuropathy- a meta-analysis
Source: Sci Rep. 2019 Nov 26;9:17549. doi: 10.1038/s41598-019-54152-8 (PMC6879539; doi:10.1038/s41598-019-54152-8)
Supplement: Supplementary file 1 — Supplementary information [file 41598_2019_54152_MOESM1_ESM.pdf]

The effect of analgesics on stimulus evoked pain-like behaviour in animal models for chemotherapy induced peripheral neuropathy- a meta-analysis

(Carlijn R Hooijmans, Derk Draper, Mehmet Ergün, Gert Jan Scheffer)

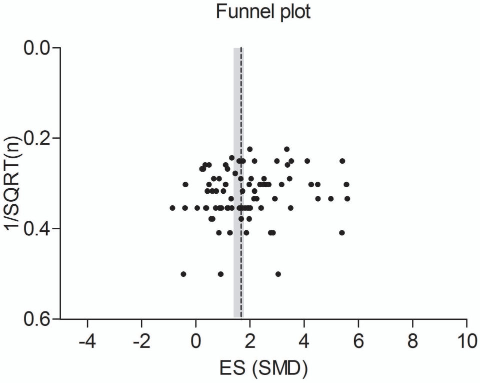

Supplemental file 1: Assessment of publication bias for the outcome mechanical evoked pain like behavior.

The panel represents the funnel plot for the outcome mechanical evoked pain like. The line represents the pooled effect estimate, the grey bands represent the global 95% confidence intervals.

The effect of analgesics on stimulus evoked pain-like behaviour in animal models for chemotherapy induced peripheral neuropathy- a meta-analysis
(Carlijn R Hooijmans, Derk Draper, Mehmet Ergün, Gert Jan Scheffer)

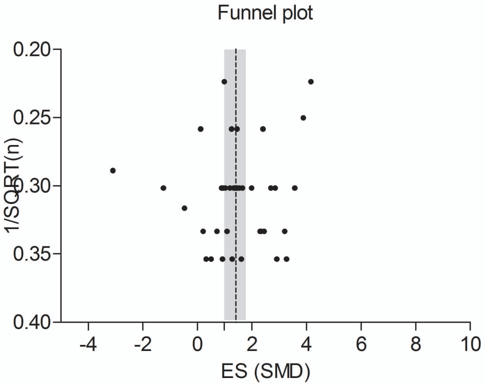

Supplemental file 2: Assessment of publication bias for the outcome cold evoked pain like behavior. The panel represents the funnel plot for the outcome cold evoked pain like behavior. The line represents the pooled effect estimate, the grey bands represent the global 95% confidence intervals
